# Allelic variation at high-molecular weight and low-molecular weight glutenin subunit genes in Moroccan bread wheat and durum wheat cultivars

**DOI:** 10.1007/s13205-017-0908-1

**Published:** 2017-08-23

**Authors:** Fatima Henkrar, Jamal El-Haddoury, Driss Iraqi, Najib Bendaou, Sripada M. Udupa

**Affiliations:** 1 International Center for Agricultural Research in the Dry Areas (ICARDA), B.P. 6299, Rabat, Morocco; 2 Biotechnology Unit, Institut National de la Recherche Agronomique (INRA), B.P. 415, Rabat, Morocco; 3 Biotechnology Laboratory, Institut National de la Recherche Agronomique (INRA), B.P. 589, Settat, Morocco; 4 Faculty of Sciences, Mohammed V University, Rabat, Morocco

**Keywords:** Moroccan wheat, Glutenin, HMW-GS, LMW-GS, PCR markers, End-use quality

## Abstract

Glutenin is a major protein fraction contributing to the functional properties of gluten and dough. The glutenin constitutes 30–40% of the protein in wheat flour and about half of that in gluten. It is essential to identify correct glutenin alleles and to improve wheat quality by selecting alleles that exert favorable effects. Moroccan wheat cultivars are unique in West Asia and North Africa region, since many of them possess resistance to Hessian fly, a pest, which is becoming important in other countries in the region. Hence, these cultivars are being used as donor for the resistance in the breeding program. Here, we determine the allelic variation in high-molecular weight glutenin subunits (HMW-GS) and low-molecular weight glutenin subunits (LMW-GS) in Moroccan cultivars of bread and durum wheat using the gene-specific PCR markers. In 20 cultivars of bread wheat, 9 different allele variants were detected at HMW-GS and 13 different allele variants were detected at LMW-GS, in which the alleles *Glu*-*A1b* (2*), *Glu*-*B1i* (17 + 18), *Glu*-*B1c* (7*/7 + 9), *Glu*-*D1d* (5 + 10)*, Glu*-*A3c, Glu*-*B3* *h,* and *Glu*-*D3b* were the most frequents. In 26 cultivars of durum wheat, less allelic variation was found: seven different allele variants at HMW-GS and six different allele variants at LMW-GS were identified, in which the major alleles were *Glu*-*A1c* (null), *Glu*-*B1b* (7 + 8), *Glu*-*B1e* (20), *Glu*-*A3c,* and *Glu*-*B3d*. The mean value of the genetic diversity for the glutenin loci was 0.502 in bread wheat and 0.449 in durum wheat. Most of the glutenin alleles carried by Moroccan bread wheat cultivars impart good bread-making quality. Most of the durum wheat glutenin alleles were related to low strength dough or poor quality and need to be improved. To improve quality of Moroccan durum wheat, essentially, *Glu*-*A1c* and *Glu*-*B3d* alleles of the genes should be replaced with the better alleles through breeding.

## Introduction

Glutenin proteins are the most important protein group which determines bread-making quality of bread wheat (*Triticum aestivum* L.) and pasta making quality of durum wheat (*Triticum turgidum* L.). It contributes to the ability of dough to rise and maintain its shape as it is baked. Glutenin strength differs with varieties of wheat. It is highly heterogeneous mixture of polymers consisting of a number of different high- and low-molecular-weight glutenin subunits (HMW-GSs and LMW-GSs) linked by disulfide bonds (Veraverbeke and Delcour 2002), resulting in variability in gluten strength among wheat varieties.

The HMW-GSs comprise about 20–30% of the glutenin (Shan et al. 2003) and play a key role in determining wheat gluten and dough elasticity. The HMW-GSs presented a high level of polymorphism. Therefore, the HMW-GSs are of immense importance in wheat breeding and genetics. A complex locus *Glu*-*1* encodes HMW-GS. *Glu*-*1* complex loci located on the long arm of chromosomes from homeologous group 1 and called *Glu*-*A1*, *Glu*-*B1,* and *Glu*-*D1* (Shewry et al. 1992). In each chromosome, the *Glu*-*1* locus contains two closely linked genes that encode for x-type glutenin subunit and y-type glutenin subunit polypeptides (Shewry et al. 1992). The LMW-GSs are quantitatively the major class of glutenin subunits which accounts for about 70–80% of the glutenins. The LMW-GS showed large effects on dough extensibility (Gianibelli et al. 2001) and gluten strength (Cornish et al. 2001) and thus influences the quality of end-use products of wheat (Gupta et al. 1990, 1991; He et al. 2005). The LMW-GSs are encoded by *Glu*-*3* loci on the short arms of homeologous group 1 and called *Glu*-*A3*, *Glu*-*B3,* and *Glu*-*D3* in bread wheat (Gupta and Shepherd 1990; Jackson et al. 1983; Masci et al. 2002). *Glu*-*3* locus is a multigene family closely linked to the *Gli*-*1* loci containing genes encoding ω and $\gamma$ gliadins.

Previous studies revealed that different alleles of HMW-GS or LMW-GS could have similar mobilities using SDS-PAGE, resulting in the incorrect identification of some alleles that are functionally different, such as Ax2 and Ax2*, Bx7 and Bx7*, By8 and By8*, Bx14–By15, and Bx20 for HMW-GS, and several alleles overlapping for LMW-GS. Hence, characterization of HMW-GS and LMW-GS genes at the DNA level and development of functional markers are needed for the discrimination of different-alleles in wheat breeding. In wheat, many functional markers are developed for the glutenin loci. The PCR-based markers are available to discriminate the important *Glu*-*1* alleles Dx5, Dy10, Ax2*, Bx7, Bx7*, Bx17, By8, and By9 (Ahmad 2000; Ma et al. 2003; Butow et al. 2004; Lei et al. 2006). Similarly, several markers are designed to differentiate the *Glu*-*3* alleles at *Glu*-*A3, Glu*-*B3,* and *Glu*-*D3* (Zhang et al. 2004; Zhao et al. 2007a, b; Wang et al. 2009).

In Morocco, several bread wheat and durum wheat cultivars have been released over the years. In recent years, bread wheat and durum wheat cultivars with the Hessian fly (Lhaloui et al. 2000, 2005) resistance have been developed and released for cultivation to tackle this pest problem in arid and semi-arid regions. Arrihane and Aguilal varieties of bread wheat were released in 1998. For durum wheat, the varieties Irden, Nassira, Chaoui, and Amria were released in 2003, Marouane in 2005, and Icamor in 2006. These resistant cultivars are useful donors for other countries in the North Africa and West Asia regions, where the Hessian fly is emerging as an important pest in recent times. However, these cultivars are not yet characterized for HMW-GS and LMW-GS variability, which is useful for marker-assisted selection in the breeding when those cultivars used as parents in the breeding program.

In Morocco, some studies were realized on the allelic variation in prolamin protein, namely, glutenin and gliadin. Using SDS-PAGE, Bakhella and Branlard (1997) observed the predominance of subunit 2*–5–17–18–10 in 44 Moroccan bread wheat cultivars and landraces, and predominance of 6–8 and 20 in 39 Moroccan durum wheat cultivars and landraces with respect to HMW-GS. In that abstract, no details regarding the landraces or cultivars used and their HMW-GS alleles were available. Zarkti et al. (2010) using also SDS-PAGE for characterization HMW-GS and LMW-GS of 23 Moroccan durum wheat landraces reported that the majority of the landraces possess the null subunit at *Glu*-*A1* and 20*x* + 20*y* at *Glu*-*B1*. However, information on HMW-GS and LMW-GS variability in the Moroccan cultivars of bread wheat and durum wheat is not available. Thus, the objective of the present study was to determine the allelic variation at *Glu*-*1* and *Glu*-*3* glutenin loci in 20 Moroccan bread and 26 Moroccan durum wheat cultivars released until 2006 using gene-specific PCR markers. The allelic information at *Glu*-*1* and *Glu*-*3* glutenin loci based on PCR-based technique, a non-destructive method, will be helpful for transferring useful alleles through genomic-assisted improvement of wheat.

## Materials and methods

### Plant materials

Total of 20 bread wheat and 21 durum wheat varieties (Table 1; Henkrar et al. 2015a, b) and 5 additional durum wheat varieties, Isly (released in 1988), Massa (released in 1988), Anouar (released in 1993), Sboula (released in 2000), and Chaoui (released in 2003) were used to characterize the glutenin alleles at *Glu*-*A1*, *Glu*-*B1*, *Glu*-*D1*, *Glu*-*A3*, *Glu*-*B3,* and *Glu*-*D3* loci. Five exotic cultivars with known glutenin subunit composition (Tables 1, 2) were used as controls to confirm the exact fragment amplified.

**Table 1:** HMW-GS composition of exotic cultivars used in this study as controls

Cultivar	*Glu*-*A1*	*Glu*-*B1*	*Glu*-*D1*	Alleles	References
Chinese-Spring		7 + 8	2 + 12	*c, b, a*	Bekes et al. (2008a)
Annuello	1	7* + 8	2 + 12	*a, u, a*	Bekes et al. (2008a)
Pavon-76	2*/1	17 + 18	5 + 10	*b/a, i, d*	Bekes et al. (2008a)
Stylet	1	7 + 9	5 + 10	*a, c, d*	Bekes et al. (2008a)
Yecora-Rojo	1	17 + 18	5 + 10	*a, i, d*	Bekes et al. (2008a)

**Table 2:** LMW-GS composition of exotic cultivars used in this study as controls

Cultivar	*GluA3*	*GluB3*	*GluD3*	References
Chinese-Spring	*a*	*a*	*a*	Bekes et al. (2008b)
Annuello	*b*	*b*	*b*	Bekes et al. (2008b)
Pavon-76	*b*	*h*	*e*?	Bekes et al. (2008b)
Stylet	*c/e*	*h*	*c*	Bekes et al. (2008b)
Yecora-Rojo	*d*	*h*	*a*	Bekes et al. (2008b)

### DNA extraction and gene-specific marker analysis

Genomic DNA was extracted from leaves at seedling stage using a CTAB (cetyltrimethylammonium bromide) protocol of Saghai-Maroof et al. (1984) with slight modification (Udupa et al. 1999). Quality and quantity of the isolated DNA were determined on 1.0% (w/v) agarose gels by comparing bands to known concentrations of lambda DNA. The PCR reactions were performed in a total volume of 10 µL, containing 1X PCR buffer (Promega, USA), 1.5 mM MgCl_2_, 200 µM of each dNTPs, 10 pmol of each primer, 0.5 U of *Taq* DNA polymerase, and approximately 50 ng of genomic DNA. All the allele-specific and gene-specific PCR primers were synthesized (Sigma-Genosys, Germany) according to published sequence information: Ax2*/Ax1/Axnull (Lafiandra et al. 1997), Ax2* (De Bustos et al. 2000), Dx5/Dx2, Dy10/Dy12, and Bx7 (Ahmad 2000), Bx/Bx7*/Bx6 (Butow et al. 2004), By8/By8*/By9/By18*/By20* (Lei et al. 2006), *Glu*-*A3* (Zhang et al. 2004), *Glu*-*B3* (Wang et al. 2009), and *Glu*-*D3* (Zhao et al. 2007a, b). The amplification programs and electrophoresis conditions of the PCR assays were based on the references mentioned above. The PCR products were separated in ethidium bromide-stained 1.2 or 1.5% (w/v) agarose gels run in 1 × TBE buffer and exposed to UV light to visualize DNA fragments.

### Statistical analysis

The gene diversity, number of alleles, and PIC value were calculated using the PowerMarker software (Ver. 3.0; Liu and Muse 2005). The glutenin relationship between cultivars was visualized as a dendrogram using the PowerMarker and MEGA5 software (Tamura et al. 2011). The Neighbor-joining tree was constructed using the frequency-based distance for the shared allele.

## Results

### Allelic variation in bread wheat cultivars

**Fig. 1:**
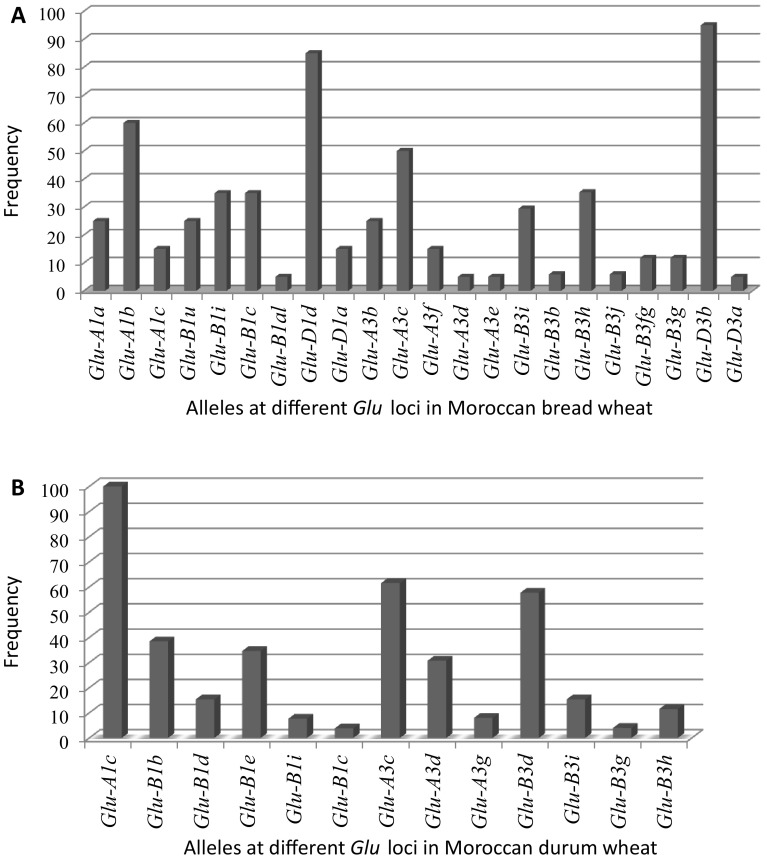
Frequency of alleles at different *Glu* loci in the 20 Moroccan bread wheat cultivars (**a**) and 26 Moroccan durum wheat cultivars (**b**)

HMW-GS and LMW-GS composition of 20 Moroccan bread wheat cultivars based on gene/allele-specific PCR analysis are shown in Table 3. The frequencies of different alleles identified were calculated and schematized in Fig. 1.

**Table 3:** HMW-GS and LMW-GS composition in Moroccan bread wheat cultivars using gene-specific PCR markers

Cultivar	HMW-GS			LMW-GS		
	*Glu*-*A1*	*Glu*-*B1*	*Glu*-*D1*	*Glu*-*A3*	*Glu*-*B3*	*Glu*-*D3*
Saïs	1 (*a*)	7*–8 (*u*)	5–10 (*d*)	*b*	*i*	*b*
Arrehane	2* (*b*)	17–18 (*i*)	5–10 (*d*)	*b*	*i*	*b*
Acsad-59	null (*c*)	7*–8 (*u*)	5–10 (*d*)	*c*	*b*	*b*
Kanz	2* (*b*)	17–18 (*i*)	5–10 (*d*)	*f*	*h*	*b*
Aguilal	1 (*a*)	7*–8 (*u*)	5–10 (*d*)	*d*	*i*	*b*
Tilila	1 (*a*)	7*–9 (*c*)	5–10 (*d*)	*c*	*j*	*b*
Achtar	2* (*b*)	17–18 (*i*)	5–10 (*d*)	*c*	*fg*	*b*
Nasma	2* (*b*)	7*–8 (*u*)	5–10 (*d*)	*c*	–	*b*
Khair	2* (*b*)	7–8* (*al*)	2–12 (*a*)	*b*	*fg*	*b*
Massira	null (*c*)	17–18 (*i*)	2–12 (*a*)	*c*	*h*	*b*
Mehdia	2* (*b*)	7*–9 (*c*)	5–10 (*d*)	*c*	*h*	*b*
Rajae	2* (*b*)	17–18 (*i*)	5–10 (*d*)	*c*	–	*b*
Amal	2* (*b*)	7*–9 (*c*)	5–10 (*d*)	*f*	*g*	*b*
Baraka	2* (*b*)	17–18 (*i*)	2–12 (*a*)	*b*	*i*	*b*
Jouda	1 (*a*)	17–18 (*i*)	5–10 (*d*)	*c*	*h*	*b*
Saba	null (*c*)	7*–9 (*c*)	5–10 (*d*)	*c*	*g*	*b*
Marchouch	2* (*b*)	7*–8 (*u*)	5–10 (*d*)	*b*	*h*	*b*
Potam	2* (*b*)	7*–9 (*c*)	5–10 (*d*)	*c*	*i*	*b*
Saada	1 (*a*)	7*–9 (*c*)	5–10 (*d*)	*f*	*h*	*a*
Salama	2* (*b*)	7*–9 (*c*)	5–10 (*d*)	*e*	–	*b*

A total of nine different allele variants were detected at HMW-GS. Three subunits (1, 2*, and null) were identified at *Glu*-*A1* locus, and the sum of the frequency of the two active types 1 (*Glu*-*A1a*) and 2* (*Glu*-*A1b*) was 85%. While the rest were null-type gene *Glu*-*A1c*. There were four subunit pairs at *Glu*-*B1* locus 7*–8 (*Glu*-*B1u*), 7–8* (*Glu*-*B1al*), 7/7*–9 (*Glu*-*B1c*), and 17–18 (*Glu*-*B1i*). Among them, the subunit pairs 7/7*–9 and 17–18 had highest proportion, 35% for each. At *Glu*-*D1* locus, the predominant HMW-GS were the combination 5–10 (*Glu*-*D1d*) at frequency of 85%. Then, 15% were for the combination 2–12 (*Glu*-*D1a*).

In LMW-GS, 13 different allele variants were identified. At *Glu*-*A3* locus, five alleles were found (*b, c, d, e,* and *i*) among which *Glu*-*A3c* occurred in 50% of the cultivars. *Glu*-*B3* appears to be highly polymorphic in this set of cultivars. Out of the six alleles (*b, fg, g, i, h,* and *j*), alleles *Glu*-*B3* *h* and *Glu*-*B3i* were predominant and showed a high frequency of 35 and 29%, respectively. The cultivars Nasma, Rajae, and Salama did show any alleles using the available allele-specific PCR markers for *Glu*-*B3*. This indicates that these cultivars had other allele types, not able to be identified using the present PCR markers and involve the SDS-PAGE technique. In addition, no allele was amplified in variety Tilila using the same set of allele-specific primers. The variety Tilila had a 1BL.1RS translocation and was derived from Veery "s" (Jlibene et al. 1996), which has been characterized to have the allele *j* (Gupta et al. 1994). Furthermore, according to Gupta et al. (1994), the allele *Glu*-*B3j* is associated with the translocated chromosome1BL.1RS. Thus, Tilila had the allele *Glu*-*B3j*. At *Glu*-*D3* locus, two alleles were identified, *Glu*-*D3a* and *Glu*-*D3b* with a frequency of 5 and 95%, respectively.

### Allelic variation in durum wheat cultivars

The HMW-GS and LMW-GS compositions of 26 Moroccan durum wheat cultivars are summarized in Table 4 and their frequencies are presented in Fig. 1. Less allelic variation was found in the Moroccan durum wheat compared to the bread wheat: six different allele variants at *Glu*-*1* (HMW-GS) and seven allele variants at *Glu*-*3* (LMW-GS). At *Glu*-*A1*, the null type was present in all cultivars studied (100%), and no active type was detected. Five alleles identified at *Glu*-*B1* loci, with subunits 6–8 (*Glu*-*B1d*), 7–8 (*Glu*-*B1b*), 7/7*–9 (*Glu*-*B1c*), 17–18 (*Glu*-*B1i*), and 20 (*Glu*-*B1e*), in which the subunit pairs 7–8 and 20 were the predominant with 38 and 35%, respectively. Among the three alleles detected at *Glu*-*A3* loci, *Glu*-*A3c* was the most frequent (62%). The *Glu*-*B3* locus exhibited four alleles (*d, i, g,* and *h*) and *Glu*-*B3d* was the major allele with high frequency of 58%. In this locus, Oum-Rabia, Tensift, and Icamor did show any allele using the available PCR markers.

**Table 4:** HMW-GS and LMW-GS composition in Moroccan durum wheat cultivars using gene-specific PCR markers

Cultivar	HMW-GS		LMW-GS	
	*Glu*-*A1*	*Glu*-*B1*	*Glu*-*A3*	*Glu*-*B3*
Karim	null (*c*)	7–8 (*b*)	*c*	*d*
Ourgh	null (*c*)	7–8 (*b*)	*c*	*d*
Oum-Rabia	null (*c*)	7–8 (*b*)	*c*	–
Sarif	null (*c*)	6–8 (*d*)	*c*	*i*
Amjad	null (*c*)	20 (*e*)	*c*	*d*
Marzak	null (*c*)	7–8 (*b*)	*d*	*d*
Jawhar	null (*c*)	20 (*e*)	*c*	*d*
Anouar	null (*c*)	7–8 (*b*)	*c*	*g*
Massa	null (*c*)	7–8 (*b*)	*c*	*h*
Isly	null (*c*)	6–8 (*d*)	*d*	*d*
Sebou	null (*c*)	17–18 (*i*)	*d*	*d*
Tensift	null (*c*)	20 (*e*)	*c*	–
Merjana	null (*c*)	7–8 (*b*)	*c*	*d*
Tomouh	null (*c*)	20 (*e*)	*c*	*d*
Tarek	null (*c*)	6–8 (*d*)	*c*	*d*
Belbachir	null (*c*)	7–8 (*b*)	*c*	*d*
Icamor	null (*c*)	20 (*e*)	*c*	–
Maroune	null (*c*)	7–8 (*b*)	*d*	*h*
Nassira	null (*c*)	7*–9 (*c*)	*c*	*d*
Chaoui	null (*c*)	20 (*e*)	*d*	*i*
Amria	null (*c*)	20 (*e*)	*d*	*i*
Cocorit	null (*c*)	6–8 (*d*)	*g*	*h*
Irden	null (*c*)	20 (*e*)	*d*	*i*
Kyperonda	null (*c*)	17–18 (*i*)	*d*	*d*
Sboula	null (*c*)	7–8 (*b*)	*c*	*d*
Selbera	null (*c*)	20 (*e*)	*g*	*d*

### Genetic diversity

**Fig. 2:**
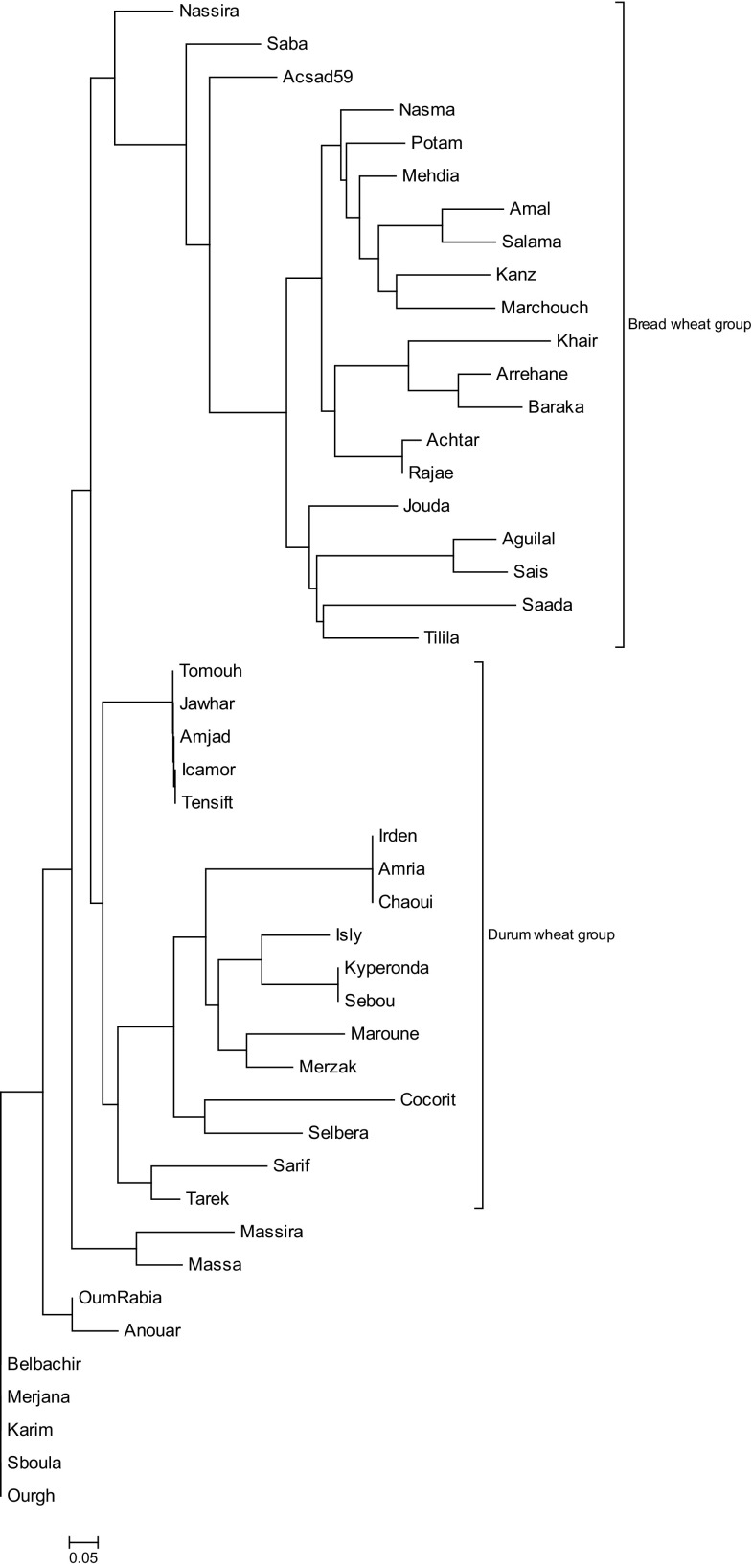
Dendrogram obtained by neighbor-joining method based on shared allele genetic distance estimates of 20 bread wheat and 26 durum wheat cultivars

The mean value of the gene diversity for the glutenin loci was 0.502 in bread wheat and 0.449 in durum wheat. Furthermore, the gene diversity of the individual loci varied widely (Table 5). The lowest value was 0.095 showed at *Glu*-*D3* locus that exhibited only two different alleles *a* and *b* in bread wheat and 0 at *Glu*-*A1* locus in durum wheat due to the overwhelming presence of the null-type gene *Glu*-*A1c*. The highest value was 0.770 at *Glu*-*B3* in bread wheat and 0.701 at *Glu*-*B1* in durum wheat. The neighbor-joining dendrogram (Fig. 2) clustered the two species in separated groups. The bread wheat cultivars were highly divergent than the durum wheat cultivars.

**Table 5:** Number of alleles, Gene diversity and PIC value of HMW-GS and LMW-GS in Moroccan bread and durum wheat cultivars

Marker	Bread wheat			Durum wheat		
	No. of alleles	Gene diversity	PIC	No. of alleles	Gene diversity	PIC
*Glu*-*A1*	3	0.555	0.491	1	0	0
*Glu*-*B1*	4	0.690	0.628	5	0.701	0.649
*Glu*-*D1*	2	0.255	0.222	–	–	–
*Glu*-*A3*	5	0.660	0.611	3	0.541	0.465
*Glu*-*B3*	6	0.754	0.717	4	0.555	0.515
*Glu*-*D3*	2	0.095	0.090	–	–	–
Mean	3.667	0.502	0.460	3.250	0.449	0.407

## Discussion

HMW-GS variations in some old varieties and landraces of bread wheat and durum wheat from Morocco were previously investigated using SDS-PAGE technique (Bakhella and Branlard, 1997). Zarkti et al. (2010) studied HMW-GS and LMW-GS variation in 23 local landraces of durum wheat using SDS-PAGE technique. The SDS-PAGE base technique is destructive and can be carried out only after the harvest of the grains and may not be handy for marker-assisted selection.

However, the HMW-GS and LMW-GS variations in the recently released bread wheat and durum wheat varieties from Morocco are lacking. Moreover, all the previous works on HMW-GS and LMW-GS variability in Moroccan wheat varieties were based on SDS-PAGE technique, which uses the harvested grains and destructive and is not useful for making selection at early stage of plant growth.

In this study, we analyzed the allelic variation of HMW-GS and LMW-GS glutenin loci in the 20 bread wheat and 26 durum wheat varieties representing the most important and recently developed cultivars in Morocco using gene/allele-specific PCR. Many of the recently developed varieties carry resistance to the Hessian fly, which is an important pest in semi-arid regions of Morocco. Because of climate change, the problem of this pest is spreading to other areas in Morocco, the North Africa and many other wheat-producing countries. The Moroccan varieties could be used as donors in wheat presumptive breeding in many counties in the semi-arid regions. Therefore, knowledge of allelic variation at *Glu*-*1* and *Glu*-*3* loci is very important for selection of suitable parents for crossing and marker-assisted selection of the Hessian resistance and better end-use quality (Henkrar et al. 2016).

Alleles present at each of the *Glu*-*1* and *Glu*-*3* loci can have a large combined effect on dough properties and suitability for specific end-products (Appelbee 2007; Eagles et al. 2006; Gupta et al. 1994). With correct classification of glutenin alleles, it is possible to improve wheat quality by selecting alleles that exert favorable effects and allelic combinations (Eagles et al. 2002). Therefore, in this study, we revealed the allelic variation of HMW-GS and LMW-GS glutenin subunit composition in 46 Moroccan wheat cultivars using PCR markers. 9 different allele variants at HMW-GS and 13 different allele variants at LMW-GS were identified in 20 cultivars of bread wheat. Six different allele variants at HMW-GS and seven allele variants at LMW-GS were noticed in 26 cultivars of durum wheat.

### Allelic variation in bread wheat cultivars

The HMW-GS composition 2* (*b*), 7/7*–9 (*c*), 17–18 (*i*), and 5–10 (*d*) was the most frequent. Odenbach and Mahgoub (1988) found that the HMW glutenin subunits 2*, 7 + 9, and 5 + 10 were associated with large sedimentation volumes. Ram (2003) reported also that the combination of *Glu*-*A1b*, *Glu*-*B1i,* and *Glu*-*D1d* alleles exhibited the highest dough strength and can be used as combination to improve bread-making quality. For *Glu*-*A1* locus, the two active types of HMW-GS 1 and 2* were detected at high frequency (85%) which appears to be a better baking quality allele and confers better values for the quality parameters than allele null (Luo et al. 2001). The same subunit had been previously described by Giraldo et al. (2010) in set of Spanish wheat landraces. Likewise, the same subunit had been found in Argentinean bread wheat (Lerner et al. 2009). However, these results are quite different to those observed in China and French bread wheat, where the allele *Glu*-*A1c* (null type) was the most frequent (Yan et al. 2007; Branlard et al. 2003).

For *Glu*-*B1* locus, four alleles were detected. The most frequent alleles were 7/7*–9 (*Glu*-*B1c*) and 17–18 (*Glu*-*B1i*). Both alleles have high sedimentation volume, but allele 17–18 (*Glu*-*B1i*) has greater effect on sedimentation and mixograph (Carrillo et al. 1990b; Ram 2003). The allele *Glu*-*B1a* which affects negatively the dough properties was not detected in our cultivars. Previous studies reported the predominance of allele 7–9 (*Glu*-*B1c*) in varieties from US, Argentina and Pakistan (Shan et al. 2007; Lerner et al. 2009; Tabasum et al. 2011). Ma et al. (2003) identified that alleles 17–18 (*Glu*-*B1i*) and 7–8 (*Glu*-*B1b*) were the major alleles in Australian wheat. In the bread wheat varieties of France and China, allele 7–8 (*Glu*-*B1b*) was the most predominant (Yan et al. 2007; Branlard et al. 2003).

At *Glu*-*D1*, Payne (1987) proved that allelic variation at *Glu*-*D1* locus had greater effects than other loci on bread-making quality. According to Gupta et al. (1989, 1994), subunit combination 5 + 10 is associated with good bread-making quality, whereas subunit combination 2 + 12 associated with poor bread-making quality. 85% of cultivars studied possessed combination 5 + 10 (*Glu*-*D1d*). Similar allelic distribution discovered in Argentinean bread wheat (Lerner et al. 2009). Nevertheless, studies on Spanish, French or Asian bread wheat (Giraldo et al. 2010; Yan et al. 2007; Terasawa et al. 2011) have reported the predominance of 2 + 12.

For LMW-GS, the *Glu*-*3* alleles have been already ranked according to their R_max_ (maximum dough resistance). The *Glu*-*A3* alleles ranked as *b* > *d* > *e* > *c*, the *Glu*-*B3* alleles ranked as *i* > *b* = *a* > *e* = *f* = *g* = *h* > *c* and the *Glu*-*D3* alleles ranked as *e* > *b* > *a* > *c* > *d* (Gupta and Shepherd 1988; Gupta et al. 1989, 1990, 1994; Gupta and MacRitchie 1994; Metakovsky et al. 1990). In the examined cultivars, the allele *Glu*-*A3c* represented 50%, and according to *R*_max_, this allele is associated with low dough resistance and ranked poor quality. Lerner et al. (2009) and Shan et al. (2007) found also similar results and predominance of allele *c* at *Glu*-*A3* locus in Argentinean and US bread wheat cultivars. At *Glu*-*B3*, the alleles *Glu*-*B3* *h* and *Glu*-*B3i* were the most frequent. The allele *Glu*-*B3i* is associated with high gluten strength, while allele *Glu*-*B3h* is related to intermediate gluten quality. Comparing the *Glu*-*B3* variation with other studies, our results is totally different to the results of US, Argentinean and French wheat in which the allele *g* was the most frequent (Shan et al. 2007; Lerner et al. 2009; Giraldo et al. 2010). The allelic variation at the *Glu*-*D3* was limited to the presence of two alleles *Glu*-*D3a* and *Glu*-*D3b*. The allele *Glu*-*D3b* was the major allele in Moroccan bread wheat (95%) and generally reported to be associated with good quality (Lerner et al. 2009). This result is similar to the results of Argentinean and US wheat (Lerner et al. 2009; Shan et al. 2007), but different to those observed in French wheat were the allele *Glu-D3* *c* was the predominant.

### Allelic variation in durum wheat cultivars

The null-type gene *Glu*-*A1c* related to less extensible or medium elastic dough (Branlard et al. 2003) was the only allele present in the 26 cultivars of durum wheat. The *Glu*-*B1b*, *Glu*-*B1e,* and *Glu*-*B1d* were predominant with 38, 35, and 15%, respectively. *Glu*-*B1b* is considered the best allele in relation to quality; *Glu*-*B1d* slightly poorer than *Glu*-*B1b* and *Glu*-*B1e* is considered the poorest (Carrillo et al. 1990a). Like in the bread wheat, the predominant allele at *Glu*-*A3* was allele c with 61% in the durum wheat cultivars of Morocco. At the *Glu*-*B3* locus, the allele *Glu*-*B3d* was the most frequent (65%) which had a medium to weak dough properties (Cornish et al. 1993; Luo et al. 2001). For the *Glu*-*A3* and *Glu*-*B3*, our results were quite different from the Spanish durum landraces (Aguiriano et al. 2008), in which they reported the predominance of allele *a* for both locus. Compared to bread wheat, durum wheat was less variable in glutenin alleles.

## Conclusion

The results obtained in this report describing the allelic compositions of Moroccan bread and durum wheat cultivars may have high allelic variability. From this analysis, two points were important. Our results obtained using PCR markers are similar to those reported previously by Bakhella and Branlard (1997) and Zarkti et al. (2010) for HMW-GS proteins in which they use SDS-PAGE. Hence, this study proves the efficiency of molecular markers to identify the correct glutenin alleles, in a non-destructive way. In general, Moroccan bread wheat cultivars carried alleles associated to good bread-making quality. However, in durum wheat cultivars, most of the alleles related to low strength dough and need to be improved. Even though many of the durum wheat cultivars and some of the bread wheat cultivars having genes for resistance to the Hessian fly could be used as donors in the breeding program, the glutinin alleles such as *Glu*-*A1c* and *Glu*-*B3d* should be avoided during selection in the breeding program.
